# The influence of flight style on the aerodynamic properties of avian wings as fixed lifting surfaces

**DOI:** 10.7717/peerj.2495

**Published:** 2016-10-20

**Authors:** John J. Lees, Grigorios Dimitriadis, Robert L. Nudds

**Affiliations:** 1 Faculty of Life Sciences, University of Manchester, Manchester, United Kingdom; 2 Department of Aerospace and Mechanical Engineering, University of Liège, Liège, Belgium

**Keywords:** Flight, Wings, Aerodynamics, Flight style, Lift, Drag

## Abstract

The diversity of wing morphologies in birds reflects their variety of flight styles and the associated aerodynamic and inertial requirements. Although the aerodynamics underlying wing morphology can be informed by aeronautical research, important differences exist between planes and birds. In particular, birds operate at lower, transitional Reynolds numbers than do most aircraft. To date, few quantitative studies have investigated the aerodynamic performance of avian wings as fixed lifting surfaces and none have focused upon the differences between wings from different flight style groups. Dried wings from 10 bird species representing three distinct flight style groups were mounted on a force/torque sensor within a wind tunnel in order to test the hypothesis that wing morphologies associated with different flight styles exhibit different aerodynamic properties. Morphological differences manifested primarily as differences in drag rather than lift. Maximum lift coefficients did not differ between groups, whereas minimum drag coefficients were lowest in undulating flyers (Corvids). The lift to drag ratios were lower than in conventional aerofoils and data from free-flying soaring species; particularly in high frequency, flapping flyers (Anseriformes), which do not rely heavily on glide performance. The results illustrate important aerodynamic differences between the wings of different flight style groups that cannot be explained solely by simple wing-shape measures. Taken at face value, the results also suggest that wing-shape is linked principally to changes in aerodynamic drag, but, of course, it is aerodynamics during flapping and not gliding that is likely to be the primary driver.

## Introduction

Birds exhibit a remarkable range of wing morphologies and flight styles. Aerodynamic theory, primarily derived from aeronautical research, broadly explains the basis for many of the observed avian wing types. Clearly, however, important differences exist between aircraft and birds. In particular, birds operate at intermediate, transitional Reynolds numbers (*Re* ≈ 10^5^) and have twisted, roughened wings composed of discreet, deformable elements (Carruthers et al., 2010). The former property makes birds of interest to the designers of unmanned air vehicles (UAVs), which operate at similar transitional *Re*. Despite broad interest regarding the aerodynamics of avian flight, however, there are relatively few comparative, empirical studies relating wing morphology to measured aerodynamic parameters.

Wings can be categorised according to their shape, which may then be related to the flight style with which their aerodynamic and inertial properties are associated (Rayner, 1988; Viscor & Fuster, 1987). Aspect ratio (*AR*, the ratio of wing span to mean chord) is a critical morphological determinant of wing performance, with large *AR* (characteristic of high speed or soaring species) reducing induced drag and providing greater maximum lift to drag ratios, particularly at slow speeds, where induced drag is relatively high (Withers, 1981). Low *AR* wings are associated with high flapping frequency, increased thrust generation and enhanced manoeuvrability during flapping flight and are accordingly characteristic of many songbirds as well as species utilising ground take-off (Drovetski, 1996). Wingtip shape indices, such as the level of pointedness and convexity and wing loading are also intimately linked to flight style (Lockwood, Swaddle & Rayner, 1998; Viscor & Fuster, 1987). For example, migrants such as ducks have high wing loading and short, narrow (high *AR*) and pointed wings associated with reduced wing inertia and induced and profile drag, suited to fast airspeeds (*U*, m s^−1^) and high flapping frequencies (Alerstam et al., 2007; Lockwood, Swaddle & Rayner, 1998). Accessory wing structures may also tune the aerodynamics of wings. For example, slotted wingtips common to soaring birds help to reduce the high induced drag requirements of low speed flight (Lockwood, Swaddle & Rayner, 1998).

Although explanations for the observed variation in avian wing morphology are well established, quantitative, comparative avian aerodynamic studies are relatively few (Dial, Heers & Tobalske, 2012; Kruyt et al., 2014; Nachtigall & Kempf, 1971; Withers, 1981). This may be in part due to the difficulty in measuring wing aerodynamics on free flying birds in the wild or in wind tunnels. Aerodynamic parameters, however, can be derived from the gliding or soaring performance of birds. Components of lift and drag are inferred from parameters such as sink speed/airspeed and glide angle (Henningsson & Hedenström, 2011; Pennycuick, 1968; Raspet, 1960; Rosen & Hedenstrom, 2001); the changing profiles of sections along the wing (Carruthers et al., 2010); the wake properties (Henningsson & Hedenström, 2011; Pennycuick et al., 1992) and pressure distributions over the wing (Usherwood, Hedrick & Biewener, 2003) at different flight velocities. Nonetheless, the continuous changes in *AR* and angle of attack in both gliding and particularly in flapping birds preclude systematic quantifications of the aerodynamic properties of wings as a function of wing-shape or position (i.e., as a fixed lifting surface). The control of wing-shape and wing position relative to the freestream can only be obtained from instrumented wings which are either static or robotically controlled (Bahlman, Swartz & Breuer, 2014). These simplified experiments are important as a foundation for understanding the more complex aerodynamics of dynamic, flapping flight across different flight groups (Spedding et al., 2008). Furthermore, testing static wings can reveal novel passive aerodynamic mechanisms, for example, the reduction of flow separation, which may be applicable to small, fixed-wing air vehicles (Álvarez et al., 2001; Bokhorst et al., 2015). Understanding the link between extant avian wing morphology and aerodynamics may also inform predictions regarding the flight capabilities of extinct species (Wang & Clarke, 2015; Wang, Mcgowan & Dyke, 2011).

Comparative aerodynamic wind tunnel data for static bird wings are few (Drovetski, 1996; Nachtigal, 1979; Nachtigall & Kempf, 1971; Nachtigall & Wieser, 1966; Reddig, 1978; Withers, 1981). Withers (1981) focused on the fixed wings of 8 bird species, ranging from swifts to hawks. Although the form of lift and drag characteristics were similar to those seen in man-made wings, normalised values of the lift and drag coefficients (*C*_lift_, *C*_drag_, respectively) were lower and higher, respectively in the bird wings. These quantitative differences can be the result of low Reynolds numbers (*Re*), rough surface quality, high flexibility and morphological differences, overall resulting in low *C*_lift_:*C*_drag_ ratios in birds compared to aeroplanes. The diversity of flight styles and wing shapes used in the study, however, and low sample numbers representing each group preclude a more detailed assessment of the relationship between morphology, flight style and static wing aerodynamics. Here, the aerodynamic properties of the fixed wings of 10 bird species comprising three broad flight style groups were determined. We tested the hypothesis that the morphologies associated with different flight styles drive differences in the static lift and drag properties of avian wings.

**Table 1:** Wing morphological measures and aerodynamic parameters of the differing flight style groups. Values are presented as the mean ± se. Wing profile colours represent the three distinct flight styles as defined in Viscor & Fuster, (1987); forward and bounding flapping flight (red), high frequency flapping flight (green) and undulating flight (purple). Letters indicate values, which are significantly different, as determined by ANOVA. Scale lines on wing outlines represent 0.1 m.

Wing profile	Species	*M*_*b*_ (kg)	*S* (m^2^)	*b*_semi_ (m)	*AR*	*C*_lift, max_	$\bar {X}$ (*C*_lift, max_)	*C*_drag, min_	$\bar {X}$ (*C*_drag, min_)	*C*_lift_:*C*_drag, max_	$\bar {X}$ (*C*_lift_:*C*_drag, max_)
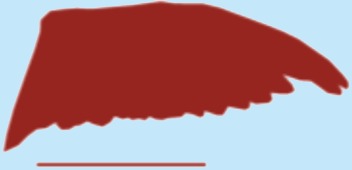	Snipe	0.11	0.021	0.19	6.88	0.77 ± 0.02	0.87 ± 0.04	0.13 ± 0.002	0.12 ± 0.005a	3.45 ± 0.04	3.47 ± 0.1a
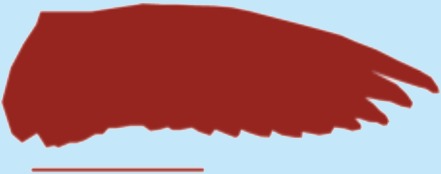	Plover	0.21	0.03	0.25	8.33	0.76 ± 0.03		0.12 ± 0.001		3.34 ± 0.2	
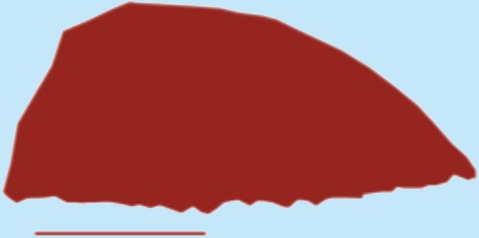	Woodcock	0.31	0.048	0.27	6.08	0.97 ± 0.05		0.13 ± 0.01		3.19 ± 0.08	
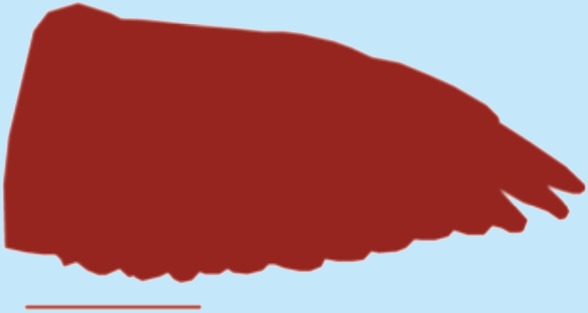	Woodpigeon	0.49	0.077	0.35	6.36	1.1 ± 0.1		0.09 ± 0.01		4.02 ± 0.2	
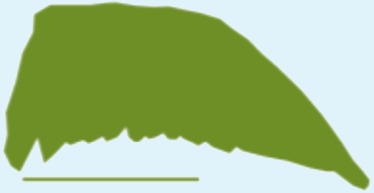	Teal	0.31	0.026	0.2	6.15	0.79 ± 0.01	0.84 ± 0.03	0.14 ± 0.002	0.11 ± 0.009b	2.76 ± 0.07	2.92 ± 0.18a,b
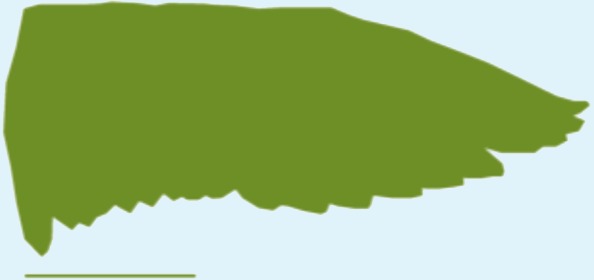	Wigeon	0.77	0.062	0.34	7.46	0.96 ± 0.04		0.1 ± 0.02		0.11 ± 0.009	
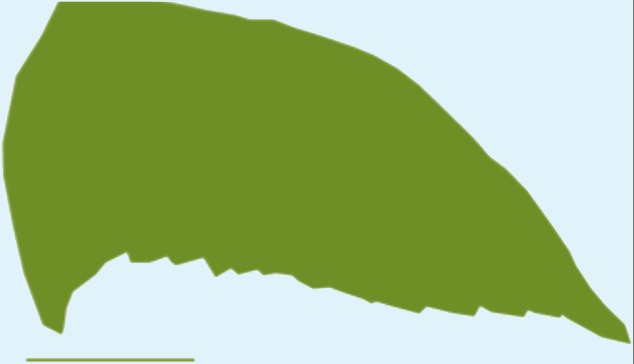	Mallard	1.08	0.087	0.35	5.63	0.8 ± 0.02		0.08 ± 0.002		2.86 ± 0.67	
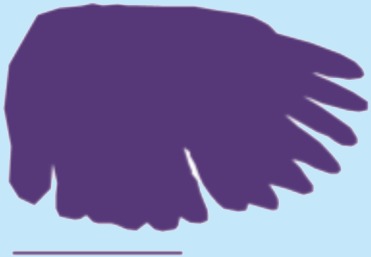	Jay	0.17	0.045	0.22	4.35	0.7 ± 0.04	0.8 ± 0.068	0.08 ± 0.003	0.08 ± 0.004a,b	3.05 ± 0.12	3.5 ± 0.12b
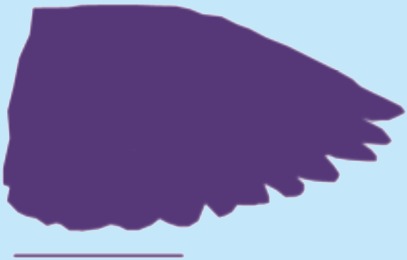	Magpie	0.21	0.05	0.25	4.97	0.69 ± 0.04		0.08 ± 0.004		3.66 ± 0.07	
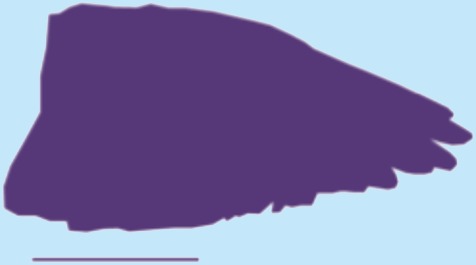	Jackdaw	0.25	0.055	0.29	6.07	1.09 ± 0.1		0.09 ± 0.01		4.01 ± 0.12	

**Notes.**

*M*_*b*_ estimated body masses (kg) S wing projected area (m^2^) *b*_semi_ wing semi-span (m) *AR* aspect ratio *C*_lift,max_ maximum lift coefficient *C*_drag,min_ minimum drag coefficient *C*_lift_:*C*_drag, max_ maximum *C*_lift_:*C*_drag_

## Materials and Methods

Wings were obtained from 10 species of bird (Table 1), including the common snipe, *Gallinago gallinago* (Linnaeus, 1758), Eurasian magpie, *Pica pica* (Linnaeus, 1758), European golden plover, *Pluvialis apricaria* (Linnaeus, 1758), jackdaw, *Corvus monedula* (Linnaeus, 1758), Eurasian jay, *Garrulus glandarius* (Linnaeus, 1758) mallard duck, *Anas platyrhynchos* (Linnaeus, 1758) teal duck, *Anas crecca* (Linnaeus, 1758), woodcock, *Scolopax rusticola* (Linnaeus, 1758) woodpigeon, *Columba palumbus* (Linnaeus, 1758) and wigeon duck, *Anas Penelope* (Linnaeus, 1758). Wings were pinned in a fully extended position and dried using borax (sodium tetraborate, minerals-water Ltd, Essex, UK). Although birds actively change wing area depending on their speed and angle of attack, a fully extended wing allows a benchmark for comparison between species. In some instances, contraction of the wings occurred during the drying process, mainly at the outermost primaries. This is an unavoidable consequence of sample preparation. It is likely, however, that the effect upon wing measures such as camber, shape and area was small and, importantly, there is no reason to expect that broad morphological differences between flight style groups were not maintained. In order to compare the aerodynamic properties of the wings in isolation, it was necessary to remove the body and rectrices of the birds prior to drying and mounting. Lift and drag data presented are thus likely to be underestimates of those in gliding birds. Although the exposed proximal ends of the prepared wings will have resulted in an increased drag compared to those smoothly faired to a bird’s body, minimum drag coefficients and lift coefficients will be minimally affected (Withers, 1981). This source of error is also consistent for all of the wings tested. Wing morphological measures, including semi span (*b*_semi_, m), and projected area (*S*, m^2^) were taken from photographs using image J (version 1.48V, US National Institutes of Health, Bethesda, MD, USA (2014)). *AR* was calculated from these measures as 2*b*_semi_^2^∕*S*. Wing pairs were attached to force/torque sensors mounted in the centre of the University of Liège Multi-Disciplinary Low Speed Wind Tunnel. This subsonic closed loop wind tunnel has a working section of 2 × 1.5 × 5 m (width × height × length) and a speed range of 2–60 m/s, with an average turbulence level of 0.15%. To obtain accurate force readings, sensors of differing sensitivity were used depending on the magnitude of forces (in turn dependent on wing size) and consisted of Nano17 and Nano25 6-axis force/torque sensors (ATI Industrial Automation, Apex, NC, USA). Lift and drag were measured at angles of attack (*α*, °) ranging from −20° to 30° in 10° increments. Wings were tested at airspeeds (*U*) of 8–16 m s^−1^, which were measured using a pitot tube. The angle of attack of the sensor was set using an electronic spirit level prior to each trial. The *α* of the wings was arbitrarily defined as the angle of the head of the sensor in the horizontal axis, which was close to *α* at mid-*b*_semi_ (when not aerodynamically loaded) as a result of the wing mounting procedure. Hence *α* only represents a relative point of comparison as the local *α* varies continuously along the wing and at different *U* as a result of aeroelastic deformations. In order to minimize dihedral or anhedral effects, the leading edge of the wing (at the propatagium perpendicular to the angle of the secondary feather rachises) was set to a horizontal position with respect to the horizontal axis of the sensor when at 0° *α*. To facilitate species comparisons, lift and drag were converted to *C*_lift_ and *C*_drag_ respectively, using:

(1) \begin{eqnarray*}{C}_{\mathrm{lift}}= \frac{\mathrm{lift}}{0.5\rho {U}^{2}S} \end{eqnarray*}

(2) \begin{eqnarray*}{C}_{\mathrm{drag}}= \frac{\mathrm{drag}}{0.5\rho {U}^{2}S} \end{eqnarray*}

where *ρ* is air density (kg m^3^). Species were placed into flight style groups following Viscor (Viscor & Fuster, 1987) for statistical comparison. These consisted of forward flapping species which primarily use a sustained horizontal flight (FF, Common snipe, European golden plover, woodcock and woodpigeon) at an elevated energetic cost, birds with high frequency flapping flight (HF, teal duck, wigeon duck and mallard duck) which is often coupled to their high respiratory frequency and is common to many aquatic and migratory species and species which utilize undulating flight (UF, jay, Eurasian magpie and jackdaw), in which there is a period of powered flight followed by a period of gliding.

### Statistics

Analysis of variance (ANOVA) was used to test for differences between flight style groups in *C*_lift_ at the *α* resulting in the maximum *C*_lift_(*C*_lift, max_), *C*_drag_ at the *α* resulting in the minimum *C*_drag_ (*C*_drag, min_) and *C*_lift_:*C*_drag_ at the *α* resulting in the maximum *C*_lift_:*C*_drag_(*C*_lift_:*C*_drag, max_). Differences in the incremental responses of *C*_lift, max_, *C*_drag, min_ and *C*_lift_:*C*_drag, max_ to *AR* and *S* were investigated using analysis of covariance (ANCOVA). The slopes and intercepts were tested for differences and where the interaction term (i.e., flight style x *AR*) was non-significant, the analysis was rerun assuming parallel lines (i.e., the interaction term was removed). Where interaction terms were significant, *post hoc* Fisher’s least significant difference procedure multiple comparison tests were employed. ANOVAs were conducted using SPSS v.22 (IBM, Somers, NY, USA) and ANCOVAs were conducted using the statistics toolbox in Matlab R2013a (The MathWorks, Inc., Natick, MA, USA). We felt that the use of phylogenetically controlled statistical analyses was not required given the relatively small sample size of the data and the fact that within flight style groups most of the species are closely related, with HF consisting of birds from the same family (Anatidae), UF consisting of birds from the same family (Corvidae) and 3 of the 4 species in the FF group consisting of birds from the same order (Charadriiformes). Therefore testing for differences between flight style groups and evolutionarily related groups would essentially be the same statistical test.

## Results

### Lift

**Figure 1:**
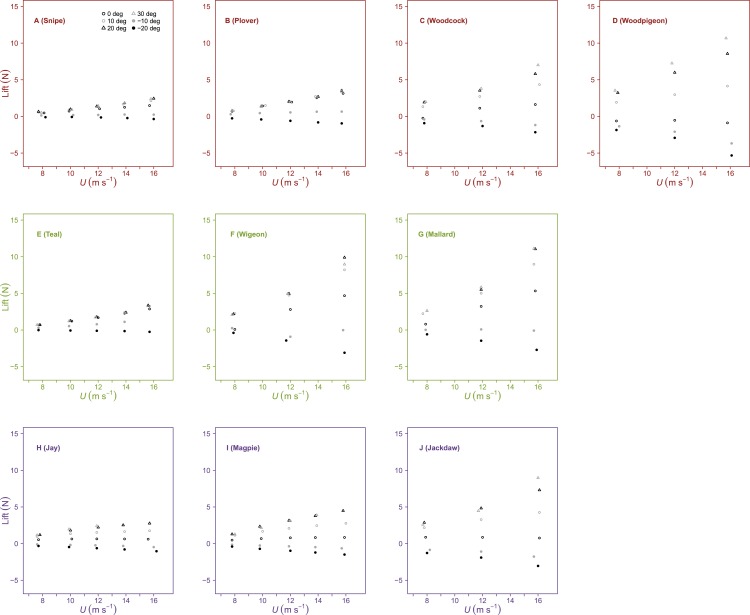
Variation in lift with airspeed and wing angle of attack. Lift (*N*) was linearly related to airspeed (*U*, m s^−1^), increasing at positive angles of attack (*α*, °) and decreasing at negative *α*. The incremental change in Lift with *U* was significantly different between species at similar values of *α*. Peak lift did not always correspond to the maximum *α*. Rows contain individuals from species of differing flight style; forward and bounding flapping flight (row 1), high frequency flapping flight (row 2) and undulating flight (row 3). Data points represent values from individual trials. Colours represent the three distinct flight styles as defined in Viscor & Fuster (1987); forward and bounding flapping flight (red), high frequency flapping flight (green) and undulating flight (purple).

Lift was influenced by both *U* and *α*, increasing linearly with *U* at positive *α* and decreasing linearly with *U* at negative *α* (Fig. 1, Tables 2 and 3). The influence of *α* upon the slope of the relationship between lift and *U* differed between species. In general, there was a significant increase in slope with *α* from 0° to 10°. Beyond these intermediate *α* interspecies differences in lift were less pronounced. In some cases, lift decreased beyond the optimal *α*, meaning peak lift did not always correspond to maximum *α*. Maximum lift increased with *S*, but did not correlate with *AR*.

**Figure 2:**
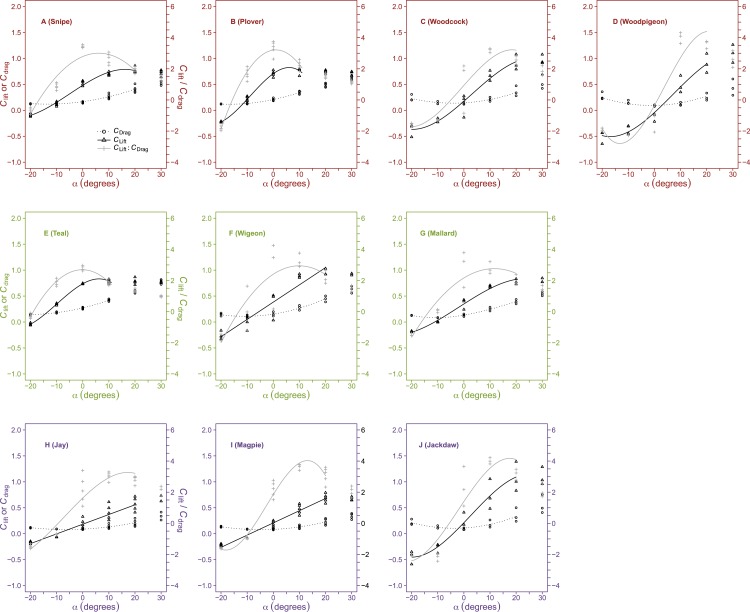
Variation in dimensionless aerodynamic parameters with wing angle of attack. The relationships between the lift coefficient (*C*_lift_, open triangles, solid black lines), drag coefficient (*C*_drag_, open circles, dotted lines) and *C*_lift_:*C*_drag_ (crosses, grey lines) and angle of attack (*α*, °) varied between species but were most commonly described by either second or third order polynomials. Maximum *C*_lift_ commonly occurred at high, positive *α* but was not different between flight style groups; forward and bounding flapping flyers (row 1), high frequency flapping flyers (row 2) and undulating flyers (row 3). Minimum *C*_drag_ occurred at intermediate *α* (0 °) and was lower in undulating flyers. The optimal *α* for *C*_lift_:*C*_drag_ varied between species and maximal *C*_lift_:*C*_drag_ values were significantly lower in the high frequency flapping flyers (anseriformes) compared to other flight style groups. Data points represent values from individual trials. Differences between groups were determined from the ANOVA. Colours represent the three distinct flight styles as defined in Viscor & Fuster (1987); forward and bounding flapping flight (red), high frequency flapping flight (green) and undulating flight (purple).

The relationship between *C*_lift_ and *α* at low and intermediate values of *α* (beyond which *C*_lift_remained relatively constant) was best fit using either linear curve fits or polynomial curves of both second and third order (Fig. 2 and Table 4).

**Figure 3:**
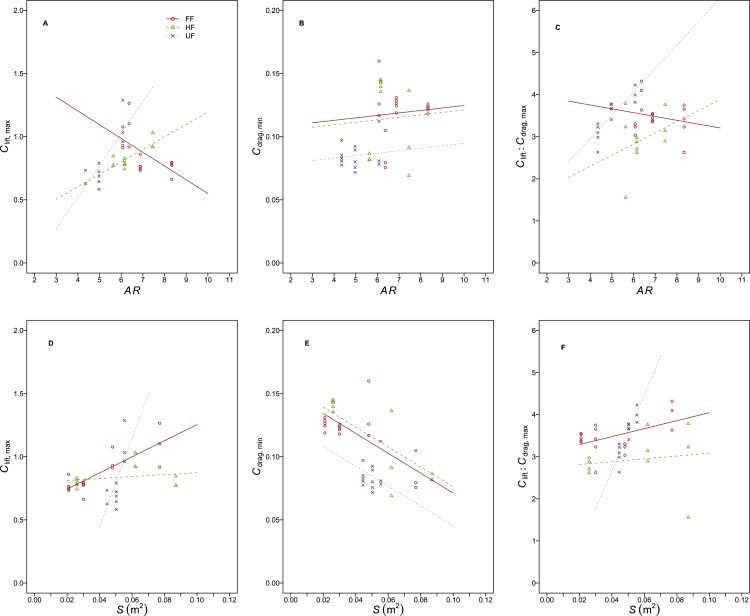
Relationships between static wing aerodynamic properties and planform morphological measures. The relationships between aerodynamic properties; lift coefficient at the maximum lift coefficient angle (*C*_lift, max_, A, D), drag coefficient at the minimum drag coefficient angle (*C*_drag_, B, E) and *C*_lift_:*C*_drag_ at the maximum *C*_lift_:*C*_drag_ angle(*C*_lift_:*C*_drag, max_, C, F) and wing morphological measures; aspect ratio (*AR*, A, B, C) and wing area (*S*, m^2^, D, E, F) differed between the different flight style groups; Forward and bounding flapping flyers (FF, red, open circles, solid lines, *N* = 4), high frequency flapping flyers (HF, green, open triangles, dashed lines, *N* = 3) and undulating flyers (UF, purple, crosses, dotted lines, *N* = 3). In particular, UF (corvids) had lower *C*_drag, min_ compared to other groups at any given *S* or *R*_*a*_. *C*_lift, max_ also increased most rapidly with *S* and *AR* in this group. Both *C*_lift, max_ and *C*_lift_: *C*_drag, max_ decreased with increasing *AR* in FF birds, a pattern not seen in the other slight style groups. Lines were determined from the ANCOVA and data points represent values from individual trials.

The incremental change in *C*_lift, max_ with *AR* differed (flight style, *F*_2,32_ = 0.19, *r*^2^ = 0.005, *p* = 0.83; *AR*, *F*_1,32_ = 0.34, *r*^2^ = 0.005, *p* = 0.56; flight style x *AR*, *F*_2,32_ = 18.8, *r*^2^ = 0.5, *p* < 0.001) between flight style groups (Fig. 3A and Table 5). A *post hoc* test showed the slope of this relationship to differ between all three flight style groups, with *C*_lift, max_ increasing with *AR* in HF and UF birds but decreasing in FF birds. The incremental change in *C*_lift, max_ with *S* differed (flight style, *F*_2,32_ = 3.06, *r*^2^ = 0.08, *p* = 0.06; *S*, *F*_1,32_ = 15.8, *r*^2^ = 0.21, *p* < 0.001 ; flight style x *S*, *F*_2,32_ = 10.9, *r*^2^ = 0.29, *p* < 0.001) between flight style groups (Fig. 3D). A *post hoc* test showed both the slope and intercept of this relationship to differ between flight style groups with the exception of the intercept of FF and HF birds, which were similar.

The incremental increase in the relationship between maximum *C*_lift_ and wing loading (*Q*, kg/m^2^) differed between flight style groups (flight style, *F*_2,32_ = 1.85, *r*^2^ = 0.08, *p* = 0.17; *Q*, *F*_1,32_ = 2.54, *r*^2^ = 0.05, *p* = 0.12; flight style x *Q*, *F*_2,32_ = 5.48, *r*^2^ = 0.22, *p* < 0.01). A *post hoc* test indicated the slope of this relationship to differ in UF birds when compared to FF and HF species, which were similar. The intercept only differed between FF and UF birds.

There was a significant effect of species upon *C*_lift, max_ (*F*_9,28_ = 11.08, *p* < 0.001). *C*_lift, max_ was highest in the woodpigeon (1.1 ± 0.1N) and jackdaw (1.1 ± 0.1N). A *post hoc* test showed *C*_lift, max_ to be significantly higher in these two species than in any other birds except the wigeon (0.96 ± 0.04N). *C*_lift, max_ however did not differ (*F*_2,35_ = 0.56, *p* = 0.57) between flight styles (Table 1).

**Table 2:** Summary of the ANCOVA analysis for the aerodynamic properties of static wings with airspeed at different angles of attack.

*α*	Dependant variable	Source	*d*.*f*.	*r*^2^	*F*	*P*
−20	Lift	Species	9	0.662	150.10	2.28E–16
		*U*	1	0.211	431.27	5.26E–15
		Species*U	9	0.117	26.50	3.26E–09
		Error	20			
	Drag	Species	9	0.587	135.00	6.44E–16
		*U*	1	0.317	655.18	9.29E–17
		Species*U	9	0.087	19.91	4.07E–08
		Error	20			
−10	Lift	Species	9	0.828	58.20	6.23E–12
		*U*	1	0.028	17.48	5.07E–04
		Species*U	9	0.114	8.01	7.79E–05
		Error	19			
	Drag	Species	9	0.436	44.78	6.58E–11
		*U*	1	0.478	441.13	1.31E–14
		Species*U	9	0.065	6.72	2.53E–04
		Error	19			
0	Lift	Species	9	0.535	242.48	2.03E–18
		*U*	1	0.223	908.54	3.82E–18
		Species*U	9	0.237	107.23	6.10E–15
		Error	20			
	Drag	Species	9	0.458	172.83	5.71E–17
		*U*	1	0.488	1659.53	1.02E–20
		Species*U	9	0.048	18.16	9.03E–08
		Error	20			
10	Lift	Species	9	0.507	205.81	1.02E–17
		*U*	1	0.347	1267.91	1.45E–19
		Species*U	9	0.141	57.32	2.54E–12
		Error	20			
	Drag	Species	9	0.546	968.26	2.15E–24
		*U*	1	0.347	5543.96	6.36E–26
		Species*U	9	0.105	186.58	2.69E–17
		Error	20			
20	Lift	Species	9	0.498	109.89	4.81E–15
		*U*	1	0.373	739.85	2.85E–17
		Species*U	9	0.119	26.14	3.68E–09
		Error	20			
	Drag	Species	9	0.543	710.23	4.72E–23
		*U*	1	0.339	3991.40	1.68E–24
		Species*U	9	0.116	151.84	2.04E–16
		Error	20			
30	Lift	Species	9	0.498	101.74	2.09E–12
		*U*	1	0.399	734.77	8.44E–15
		Species*U	9	0.094	19.29	5.71E–07
		Error	16			
	Drag	Species	9	0.523	403.40	3.95E–17
		*U*	1	0.372	2582.50	4.07E–19
		Species*U	9	0.102	78.56	1.56E–11
		Error	16			

**Notes.**

*U* airspeed (m s^−1^), Lift and Drag are presented in N

**Table 3:** Summary of the regressions from the ANCOVA analysis of static wing aerodynamic properties with airspeed at different angles of attack. Regressions are presented as *Y* = *bU* + *a*, where *Y* is the dependant variable, *a* is a constant, *b* is the regression slope and *U* is airspeed (m s^−1^).

		Dependant variable			
		Lift (N)		Drag (N)	
Species	*α*	a	b	a	b
Snipe	−20	0.22	−0.03	−0.25	0.04
	−10	0.04	0.01	−0.17	0.04
	0	−0.59	0.13	−0.16	0.04
	10	−1.01	0.20	−0.17	0.05
	20	−1.10	0.22	−0.33	0.09
	30	−1.42	0.24	−0.66	0.14
Plover	−20	0.46	−0.09	−0.31	0.05
	−10	−0.02	0.05	−0.26	0.05
	0	−1.64	0.30	−0.27	0.07
	10	−2.15	0.36	−0.56	0.12
	20	−1.90	0.33	−0.84	0.18
	30	−1.91	0.33	−1.18	0.24
Woodcock	−20	0.36	−0.15	−0.37	0.11
	−10	0.39	−0.10	−0.33	0.08
	0	−1.87	0.23	−0.29	0.07
	10	−1.45	0.36	−0.37	0.10
	20	−1.96	0.48	−0.36	0.15
	30	−3.16	0.62	−0.67	0.24
Woodpigeon	−20	1.63	−0.42	−0.65	0.20
	−10	1.10	−0.29	−0.47	0.13
	0	−0.30	−0.03	−0.35	0.08
	10	−0.27	0.28	−0.25	0.09
	20	−2.12	0.68	−0.37	0.17
	30	−3.32	0.89	0.13	0.21
Teal	−20	0.21	−0.03	−0.29	0.05
	−10	−0.73	0.13	−0.23	0.05
	0	−1.51	0.27	−0.42	0.09
	10	−1.80	0.31	−0.68	0.14
	20	−1.99	0.33	−1.08	0.21
	30	−1.29	0.26	−1.05	0.24
Wigeon	−20	2.38	−0.34	−1.15	0.17
	−10	0.24	−0.04	−0.47	0.09
	0	−4.45	0.58	−0.14	0.08
	10	−3.94	0.75	−0.63	0.18
	20	−5.80	0.96	−1.32	0.32
	30	−4.73	0.85	−2.02	0.46
Mallard	−20	1.62	−0.27	−0.99	0.17
	−10	0.16	−0.01	−0.63	0.11
	0	−3.69	0.57	−0.37	0.11
	10	−4.47	0.84	−0.92	0.24
	20	−6.46	1.08	−1.81	0.41
	30	−6.53	1.10	−2.91	0.60
Jay	−20	0.35	−0.08	−0.37	0.07
	−10	0.25	−0.04	−0.33	0.06
	0	0.52	0.01	−0.24	0.05
	10	0.33	0.09	−0.02	0.04
	20	−0.24	0.20	0.08	0.06
	30	−1.05	0.30	0.03	0.08
Magpie	−20	0.62	−0.13	−0.42	0.09
	−10	0.34	−0.06	−0.34	0.06
	0	0.20	0.04	−0.20	0.05
	10	−0.40	0.20	−0.07	0.05
	20	−1.62	0.39	−0.05	0.08
	30	−2.25	0.44	−0.34	0.14
Jackdaw	−20	0.58	−0.22	−0.38	0.12
	−10	0.22	−0.12	−0.30	0.07
	0	0.98	−0.01	−0.22	0.05
	10	0.26	0.25	−0.01	0.07
	20	−1.38	0.53	0.004	0.13
	30	−3.77	0.77	−0.44	0.25

**Notes.**

*α* angle of attack (°)

### Drag

**Figure 4:**
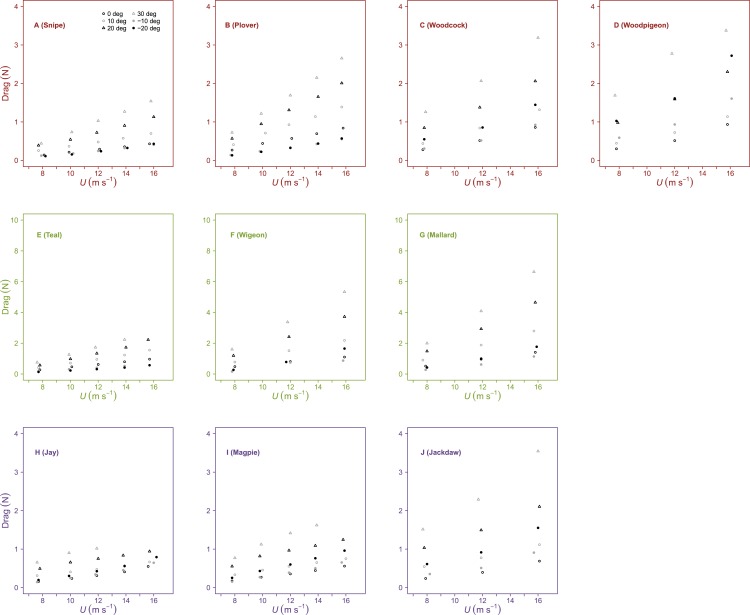
Variation in drag with airspeed and wing angle of attack. Drag (*N*) increased linearly with airspeed (*U*, m s^−1^) at all angles of attack (*α*, °). The incremental change in drag with *U* was higher at larger values of *α* and significantly different between species at similar values of *α*. Rows contain individuals from species of differing flight style as defined in Viscor & Fuster (1987); forward and bounding flapping flight (row 1, red outline), high frequency flapping flight (row 2, green outline) and undulating flight (row 3, purple outline).

Drag increased with *U* at all *α* and, in general, drag increased with less negative values of *α* at all values of *U* (Fig. 4, Tables 2 and 3). The slope of the relationship between drag and *U* increased with increasing *α*. Minimum values of drag increased with *S* but were not correlated with *AR*.

**Table 4:** Summary of the regression analyses for the aerodynamic properties of static bird wings as a function of their angle of attack. Regressions are presented as *Y* = *a* + *bα* + *cα*^2^ + *dα*^3^, where *a* is a constant and *b*, *c* and *d* are the first, second and third order terms, respectively. Higher order terms are omitted where they did not improve the model fit further.

Species		*C*_lift_ = *a* + *bα* + *cα*^2^ + *dα*^3^	*F*	*r*^2^	*P*	*C*_drag_ = *a* + *bα* + *cα*^2^ + *dα*^3^	*F*	*r*^2^	*P*	*C*_lift_:*C*_drag_ = *a* + *bα* + *cα*^2^ + *dα*^3^	*F*	*r*^2^	*P*
Snipe	(a)	4.81E–01	356.4	0.98	<0.001	1.67E–01	108.4	0.90	<0.001	2.79E+00	92.4	0.88	<0.001
	(b)	3.15E–02				6.95E–03				7.04E–02			
	(c)	−3.69E–04				2.54E–04				−5.72E–03			
	(d)	−2.50E–05				–				–			
Plover	(a)	7.07E–01	488.0	0.99	<0.001	2.08E–01	222.0	0.96	<0.001	3.20E+00	333.2	0.97	<0.001
	(b)	3.57E–02				1.07E–02				2.30E–02			
	(c)	−2.20E–03				3.15E–04				−1.16E–02			
	(d)	−8.27E–05				–				–			
Woodcock	(a)	1.75E–01	98.2	0.94	<0.001	1.44E–01	36.7	0.81	<0.001	9.34E–01	29.0	0.83	<0.001
	(b)	4.41E–02				2.89E–03				1.90E–01			
	(c)	2.75E–04				3.52E–04				−4.89E–04			
	(d)	−2.87E–05				–				−1.68E–04			
Woodpigeon	(a)	−4.27E–02	114.5	0.95	<0.001	9.38E–02	24.6	0.74	<0.001	−3.56E–01	45.3	0.89	<0.001
	(b)	4.64E–02				8.27E–04				3.04E–01			
	(c)	6.16E–04				4.15E–04				4.10E–03			
	(d)	−2.97E–05				–				−3.71E–04			
Teal	(a)	7.37E–01	1657.0	1.00	<0.001	2.72E–01	438.6	0.98	<0.001	2.69E+00	430.5	0.98	<0.001
	(b)	2.79E–02				1.20E–02				−2.46E–03			
	(c)	−1.71E–03				2.74E–04				−7.66E–03			
	(d)	−5.77E–05				–				–			
Wigeon	(a)	3.84E–01	126.0	0.90	<0.001	1.52E–01	73.2	0.91	<0.001	2.40E+00	17.9	0.71	<0.001
	(b)	3.33E–02				7.79E–03				1.09E–01			
	(c)	–				3.58E–04				−5.52E–03			
	(d)	–				–				–			
Mallard	(a)	3.48E+00	270.3	0.98	<0.001	1.33E–01	210.3	0.97	<0.001	2.16E+00	44.0	0.83	<0.001
	(b)	3.22E–02				8.64E–03				1.03E–01			
	(c)	−9.11E–05				3.19E–04				−4.40E–03			
	(d)	−1.66E–05				−5.02E–06				–			
Jay	(a)	1.83E–01	297.0	0.92	<0.001	8.82E–02	59.8	0.81	<0.001	1.62E+00	81.4	0.90	<0.001
	(b)	1.87E–02				2.42E–03				1.62E–01			
	(c)	–				1.91E–04				−2.12E–03			
	(d)	–				–				−1.00E–04			
Magpie	(a)	2.08E–01	582.4	0.96	<0.001	8.30E–02	44.0	0.78	<0.001	1.85E+00	249.3	0.97	<0.001
	(b)	2.36E–02				2.31E–03				2.69E–01			
	(c)	–				2.45E–04				−2.89E–03			
	(d)	–				–				−3.78E–04			
Jackdaw	(a)	2.11E–01	63.1	0.92	<0.001	1.10E–01	26.9	0.75	<0.001	1.40E+00	31.0	0.84	<0.001
	(b)	5.26E–02				2.94E–03				2.50E–01			
	(c)	2.73E–04				4.05E–04				−1.40E–03			
	(d)	3.51E–05				–				−2.18E–04			

**Notes.**

*C*_lift_ lift coefficient *C*_drag_ drag coefficient

**Table 5:** Summary of the ANCOVA analysis for the aerodynamic properties of static wings with morphological measures.

		Full model				Minimum adequate model				Flight style groups					
Dependant variable	Source	*d*.*f*.	*r*^2^	*F*	*P*	*d*.*f*.	*r*^2^	*F*	*P*	FF		HF		UND	
										Intercept	Slope	Intercept	Slope	Intercept	Slope
*C*_lift, max_	Flight style	2	0.01	0.19	0.83	*	*	*	*						
	*AR*	1	0.01	0.34	0.56	*	*	*	*	1.64	−0.11	0.21	0.1	−0.48	0.25
	Flight style x *AR*	2	0.53	18.77	0.00	*	*	*	*						
	Error	32				*									
	Flight style	2	0.08	3.06	0.06	*	*	*	*						
	*S*	1	0.21	15.83	3.72E–04	*	*	*	*	0.62	6.39	0.8	0.78	−0.48	0.25
	Flight style x *S*	2	0.29	10.91	2.44E–04	*	*	*	*						
	Error	32				*									
*C*_drag, min_	Flight style	2	0.16	3.34	0.05	2.00	0.16	3.52	0.04						
	*AR*	1	0.00	0.18	0.67	1.00	0.00	0.19	0.66	0.11	0.002	0.1	0.002	0.08	0.002
	Flight style x *AR*	2	0.01	0.11	0.89	*	*	*	*						
	Error	34				36.00									
	Flight style	2	0.30	17.14	7.10E–06	2.00	0.30	16.33	8.99E–06						
	*S*	1	0.37	41.52	2.30E–07	1.00	0.37	39.54	2.87E–07	0.15	−0.79	0.16	−0.79	0.12	−0.79
	Flight style x *S*	2	0.03	1.90	0.16	*	*	*	*						
	Error	34				36.00									
*C*_lift_:*C*_drag, max_	Flight style	2	0.26	8.11	1.31E–03	*	*	*	*						
	*AR*	1	0.04	2.65	0.11	*	*	*	*	4.12	−0.09	1.24	0.26	0.74	0.56
	Flight style x *AR*	2	0.15	4.71	0.02	*	*	*	*						
	Error	34				*									
	Flight style	2	0.27	8.86	7.97E–04	*	*	*	*						
	*S*	1	0.07	4.84	0.03	*	*	*	*	3.1	9.48	2.75	3.39	−0.95	90.47
	Flight style x *S*	2	0.15	5.10	0.01	*	*	*	*						
	Error	34				*									

**Notes.**

*AR* aspect ratio *S* wing area (m^2^) *C*_lift, max_ lift coefficient at the angle of attack resulting in the maximum lift coefficient *C*_drag, min_ drag coefficient at the angle of attack resulting in the minimum drag coefficient *C*_lift_:*C*_drag, max_ *C*_lift_:*C*_drag_ at the angle of attack resulting in the maximum *C*_lift_:*C*_drag_

*C*_drag_ changed curvilinearly with *α*, decreasing slightly in magnitude from *α* =  − 20° to 0° and increasing at higher *α* (Fig. 2 and Table 4).

The incremental change in *C*_drag, min_ with *AR* was not different (flight style x *AR*, *F*_2,34_ = 0.11, *r*^2^ = 0.01, *p* = 0.89) between flight style groups (Fig. 3B and Table 5). Furthermore, *C*_drag, min_ was not affected by *AR* but differed between flight style groups (flight style, *F*_2,36_ = 3.5, *r*^2^ = 0.16, *p* < 0.05; *AR*, *F*_1,36_ = 0.19, *r*^2^ = 0.004, *p* = 0.66). A *post hoc* test suggested that UF birds have a lower *C*_drag, min_ than the HF group (mean difference = 0.03, 95% CI [0.0046–0.0482]) and the FF species (mean difference = 0.03, 95% CI [0.005–0.055]). The incremental change in *C*_drag, min_ with *S* did not differ (flight style x *S*, *F*_2,34_ = 1.9, *r*^2^ = 0.03, *p* = 0.16) between flight style groups (Fig. 3E and Table 5). *C*_drag, min_ decreased with *S* (common slope = − 0.79) and differed between flight style groups (flight style, *F*_2,36_ = 16.33, *r*^2^ = 0.3, *p* < 0.001; *S*, *F*_1,36_ = 39.54, *r*^2^ = 0.36, *p* < 0.001). A *post hoc* test showed UF birds to be lower in terms of *C*_drag, min_ than HF birds (mean difference = 0.03, 95% CI [0.002–0.2]) but not FF birds.

The incremental change in minimum*C*_drag_ with *Q* was different between flight style groups, and was negative in FF and HF birds but positive in UF species (flight style, *F*_2,34_ = 13.78, *r*^2^ = 0.32, *p* < 0.001; *Q*, *F*_1,34_ = 3.57, *r*^2^ = 0.04, *p* = 0.07; flight style x *Q*, *F*_2,34_ = 10.24, *r*^2^ = 0.24, *p* < 0.001). A *post hoc* test showed a difference in the slope and intercept of this relationship between HF birds compared to the other two style groups, which were similar.

There was a significant effect of species upon *C*_drag, min_ (*F*_9,30_ = 12.94, *p* < 0.001). The lowest *C*_drag, min_ values were found in the jay (0.085 ± 0.003), magpie (0.082 ± 0.004), mallard (0.083 ± 0.002), woodpigeon (0.087 ± 0.009) and jackdaw (0.09 ± 0.011). A *post hoc* test showed that these values were not significantly different but were significantly lower than in all species except the wigeon and woodpigeon. The highest value of *C*_drag_ was found in the teal duck (0.14 ± 0.002). A post hoc test showed that this value was higher than in any other species with the exception of the snipe and woodcock. *C*_drag, min_ (Table 1) was significantly influenced by flight style (*F*_2,37_ = 10.09, *p* < 0.001). A *post hoc* test showed both the FF (0.12 ± 0.005) and HF (0.11 ± 0.009) groups to have higher *C*_drag, min_ than that of the UF group (0.08 ± 0.003). *C*_drag_ at the *α* corresponding to the maximum *C*_lift_:*C*_drag_ was significantly different between species (*F*_9,30_ = 11.18, *p* < 0.001) and between flight style groups (*F*_2,37_ = 8.14, *p* < 0.01). Mean *C*_drag_ values were highest in the HF species (0.23 ± 0.02) and lowest in the UND species (0.14 ± 0.01).

### Lift:Drag

Peak *C*_lift_:*C*_drag_ was recorded at *α* = 0° and 10° (Fig. 2) and values decreased above or below this optimal *α*, with negative values at the lowest *α* (−20°).

The incremental change in *C*_lift_:*C*_drag, max_ with *AR* (Fig. 3C and Table 5) differed between flight style groups (flight style, *F*_2,34_ = 8.11, *r*^2^ = 0.26, *p* < 0.01; *AR*, *F*_1,34_ = 2.65, *r*^2^ = 0.04 *p* = 0.11; flight style x *AR F*_2,34_ = 4.71, *r*^2^ = 0.15, *p* < 0.05). A *post hoc* test showed the slope and intercept of this relationship to differ only between FF and UF species.

The incremental change in *C*_lift_:*C*_drag, max_ with *S* (Fig. 3F and Table 5) was positive and different between flight style groups (flight style, *F*_2,34_ = 8.86, *r*^2^ = 0.27, *p* < 0.001; *S*, *F*_1,34_ = 4.84, *r*^2^ = 0.07, *p* < 0.05; flight style x *SF*_2,34_ = 5.1, *r*^2^ = 0.15, *p* < 0.05). A *post hoc* test showed the slope of this relationship to be steeper in UF birds compared to the other style groups, which were similar. Similarly, the intercept value was lower for UF species compared to those of HF or FF birds.

The incremental change in peak *C*_lift_:*C*_drag_ with *Q* was different between flight style groups (flight style, *F*_2,34_ = 1.51, *r*^2^ = 0.06, *p* = 0.24; *Q*, *F*_1,34_ = 0.76, *r*^2^ = 0.02 *p* = 0.39; flight style x *QF*_2,34_ = 5.41, *r*^2^ = 0.22, *p* < 0.01). A *post hoc* test showed the slope and intercept of this relationship to differ only between FF and UF species.

There was an effect of species upon *C*_lift_:*C*_drag, max_ (*F*_9,30_ = 4.34, *p* < 0.01), which was highest in the woodpigeon (4 ± 0.2) and Jackdaw (4 ± 0.12). These values were significantly different from the lowest values of *C*_lift_:*C*_drag, max_ seen in the teal (2.76 ± 0.07) and the mallard (2.86 ± 0.67). *C*_lift_:*C*_drag, max_ (Table 1) was also influenced by flight style (*F*_2,37_ = 5.6, *p* < 0.01). A *post hoc* test showed HF birds to have significantly lower maximum values of *C*_lift_:*C*_drag, max_ (2.92 ± 0.18) than FF (3.47 ± 0.1) and UF (3.5 ± 0.12) birds, which were similar.

## Discussion

Here we present the first statistically validated quantitative assessment of the aerodynamic properties of static wings from birds that differ in their flight styles The findings suggest that the differences in morphology associated with differing flight styles manifest only in differences in drag and not lift performance as *C*_drag, min_ but not *C*_lift, max_ differed between flight style groups. Differences between flight style groups in the incremental responses of aerodynamic parameters to morphological measures, however, indicate that general wing-morphological measures based upon planform cannot alone predict the gliding performance of avian wings as fixed lifting surfaces.

**Figure 5:**
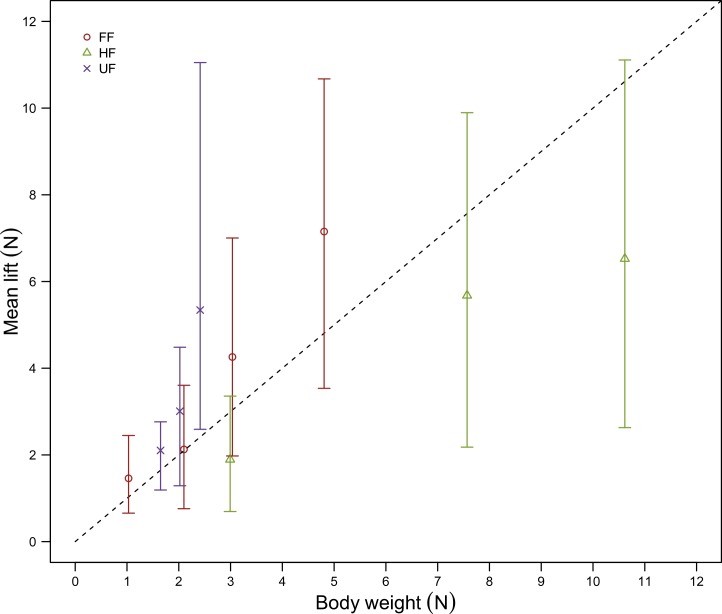
Mean lift in comparison to body weight. Mean values of lift (*N*,  ± maximum and minimum values) at the angle of attack corresponding to the maximum mean lift value (at airspeeds between 8 and 16 m s^−1^) were of sufficient magnitude to support body weight (*N*, indicated by the dashed line) across all flight style groups; Forward and bounding flapping flyers (FF, red open circles, *N* = 4), high frequency flapping flyers (HF, green open triangles, *N* = 3) and undulating flyers (UF, purple crosses, *N* = 3). In HF species, however, body support could was only achieved at the highest airspeeds. Body weights were estimates from Dunning (1993).

The lift values recorded were realistic relative to the *M*_*b*_ of the species from which the wings were taken. Mean lift values at the optimal angle of attack (with respect to lift) were sufficient to support the body weight of the birds (Fig. 5). In order to facilitate statistical comparison of the different species based upon the influence of wing morphology alone, it was necessary to remove the confounding effects of the bird’s body and rectrices . (Maybury & Rayner, 2001). In reality, however, lift values of the whole bird would be even higher, taking into account the extra component of lift generated by these features. The three species in which mean lift values fell below those required to support body weight over much of the airspeed range are the members of the Anatidae (teal duck, wigeon duck and mallard), which is unsurprising given the high *Q* characteristic of species with high-speed wings such as ducks. The duck wings have relatively low *S* and rely on high frequency high speed flapping flight at high *U* (Savile, 1957). Teal, wigeon and mallard ducks fly at speeds of around 19.7, 20.6 and 18.5 m/s (Alerstam et al., 2007), explaining why lift values only exceeded body weight at the highest airspeeds tested.

*C*_lift_ values were similar to those reported previously for both static wings (Nachtigall & Kempf, 1971; Reddig, 1978; Withers, 1981) and for birds flying in a wind tunnel (Parrott, 1970; Pennycuick, 1968). Withers (1981) obtained a maximal *C*_lift_ value of 0.9 at an *α* of 15° in dried woodcock wings and overall had a maximum *C*_lift_ range of 0.8–1.15. Similarly our mean *C*_lift_ was 0.97 at the *α* corresponding to *C*_lift, max_ of 30° in the woodcock and values across the 10 species ranged from 0.69 to 1.1. These are within the expected range for similarly cambered rigid wings such as the Eppler 387 at similar *Re* (∼70 × 10^3^) (Spedding et al., 2008). Unlike these rigid wings however, the lift:drag polars of the bird wings here did not show any abrupt step changes. This may, in part, be the result of the flexibility of the feathers which delay the onset of flow separation by reducing the angle of attack as *Re* increases. Feather roughness also reduces flow separation, further improving the performance of the wing (Bokhorst et al., 2015).

**Figure 6:**
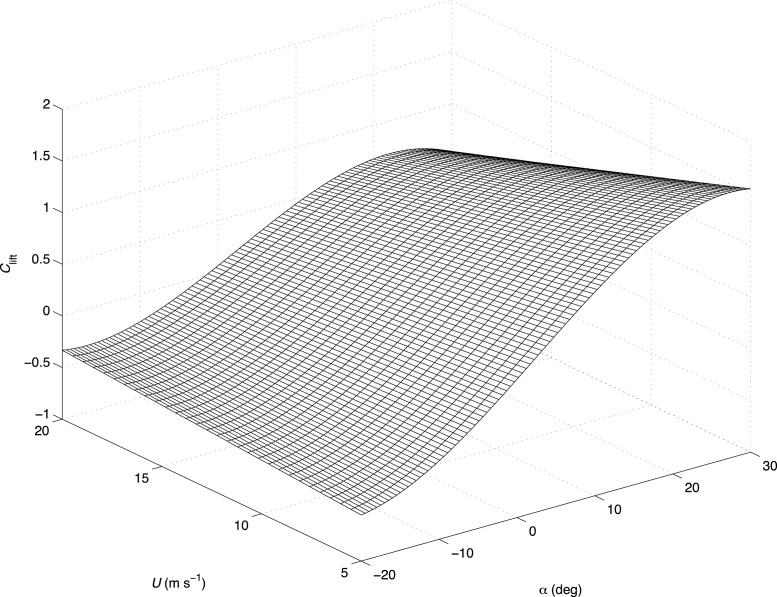
Example of a non-linear curve fit of the lift coefficient, *C*_lift_ with airspeed and angle of attack. As a result of the changing conformation of the wings at any combination of angle of attack (*α*, °) and airspeed (*U*, m s^−1^), lift and drag properties (such as *C*_lift_) of avian wings may be best visualised using a two-dimensional nonlinear curve (here shown for the jackdaw).

In classical aerodynamics, the *C*_lift_ is nearly constant with airspeed, so that the lift increases quadratically with airspeed. Here it increases curvi-linearly with airspeed (Fig. 2). This is likely to result from the flexibility of bird wings, which results in a unique wing conformation for any given value of *α* and *U*. Although for statistical comparisons, it was necessary to compare wings at their optimal *α* with respect to *C*_lift_, when evaluating the aerodynamic properties of static avian wings as a whole, *C*_lift_ (and *C*_drag_) may also be visualised as a two-dimensional nonlinear curve fit (Fig. 6) of the form:

\begin{eqnarray*}{C}_{\mathrm{lift}}={C}_{\mathrm{lift0}}+{C}_{\mathrm{lift1}}U+{C}_{\mathrm{lift2}}\alpha +{C}_{\mathrm{lift3}}\alpha U+{C}_{\mathrm{lift4}}{\alpha }^{2}+{C}_{\mathrm{ lift5}}{\alpha }^{3} \end{eqnarray*}

\begin{eqnarray*}{C}_{\mathrm{Drag}}={C}_{\mathrm{Drag0}}+{C}_{\mathrm{Drag1}}U+{C}_{\mathrm{Drag2}}\alpha +{C}_{\mathrm{Drag3}}\alpha U+{C}_{\mathrm{Drag4}}{\alpha }^{2}+{C}_{\mathrm{ Drag5}}{\alpha }^{3}. \end{eqnarray*}

Despite a variety of flight styles, *C*_lift, max_ did not differ between flight style groups. This is in agreement with previous findings for static wings (Withers, 1981) and also with studies of rotating wings, in which *C*_lift_ is more representative of that of a continuous downstroke (Dial, Heers & Tobalske, 2012; Usherwood & Ellington, 2002). Wing shape evolution is driven by a combination of aerodynamic and ecological selection pressures, modified by the constraints of phylogeny. Flapping flight involves complex conformational changes in the wing, in which pitch and span are continuously varying and the wing tip travels faster than the root. The different kinematic and aerodynamic demands of flapping and gliding mean that wings cannot be optimised for both. Wings may therefore be ‘tuned’ towards optimal performance in one flight style or the other. For example, the tuning of wing morphology to flapping as opposed to gliding has been demonstrated in swifts which show higher span efficiency but higher total drag during flapping (Henningsson, Hedenström & Bomphrey, 2014). Here, the similarities in lift performance of morphologically diverse static wings, suggest that specialisations to different flapping flight styles result primarily in difference in drag when wings are in a gliding position. Additionally, the observed *C*_lift, max_ values may be tightly constrained within the range observed, at the expense of drag for any given wing shape. Withers (1981) proposed a correlation between the lift and drag performance of bird wings, in which low profile drag coefficients (*C*_drag, pro_) were associated with low maximum *C*_lift_ but high *C*_lift_:*C*_drag_. The lack of any significant differences in*C*_lift, max_ between flight style groups here suggest that any differences in *C*_lift_:*C*_drag_ result primarily from differences in *C*_drag_ and are not strongly influenced by such a trade-off.

*C*_drag_ values were similar to those previously measured for static avian wings with the exception of the low values reported for swifts (*Apus apus* (Linnaeus, 1758)) (∼0.03) (Withers, 1981). These birds might be expected to have lower values than the species tested here as a result of their exceptional gliding capabilities. Drag data for free-flying birds or those in a wind tunnel are biased towards soaring or gliding species (Henningsson & Hedenström, 2011; Parrott, 1970; Raspet, 1960; Tucker, 1987). As with other static wing data, *C*_drag_ is lower in these species than in our wings, but their upper values fall within our *C*_drag_ envelope. For example, our *C*_drag, min_ values ranged from 0.8 to 0.14 which is at the upper end of the values for the swift and vulture (Henningsson & Hedenström, 2011; Parrott, 1970). Pigeons flying at 10 m s^−1^, however have estimated *C*_drag_ values of around 0.25 (approximated using both wing and tail area), which is higher than any of the values for species tested here (Pennycuick, 1968). Although drying and mounting wings separate from the birds’ bodies is likely to elevate their drag characteristics to some extent, the similarities between both our *C*_lift_ and *C*_drag_ values and those of free-flying birds indicate that this effect is minimal during gliding flight. One marked difference, however, between our wings and those of free-flying birds is the constant wingspan throughout the range of flight speeds. With the apparent exception of the black vulture, free flying birds actively reduce their wingspan, area and therefore aspect ratio with increasing airspeeds, reducing profile drag and maintaining a moderate *C*_lift_ (Henningsson & Hedenström, 2011; Parrott, 1970; Pennycuick, 1968).

Unlike *C*_lift, max_, *C*_drag, min_ differed between the different flight style groups and was significantly lower in the undulating flyers. Undulating flight, common to corvid species involves periods of flapping flight in order to gain height, separated by bouts of gliding flight. As *C*_lift_ appears unaffected by wing shape in gliding, it seems that selection has driven aerodynamic adaptations towards reducing drag in species that rely on phases of gliding flight. In the corvids, low *C*_drag, min_ values, in addition to *C*_drag_ values at *α* corresponding to the maximum *C*_lift_:*C*_drag_ may, in part be explained by the slotting of their wing tips. Slotting is associated with primary feathers that are separated both horizontally and vertically in flight, spreading vorticity and reducing induced drag (Tucker, 1993). Pertinently, the separation of feathers characteristic of wing slotting was observed in the wind tunnel in UF species but not in HF or FF species. Although the minimum and maximum values of aerodynamic parameters were chosen as reference points for the purposes of statistical comparison, some of the species tested will fly at combinations of *α* and *U* which result in values of lift and drag outside of these values. It is possible that observed values of *C*_lift, max_, *C*_drag, min_ and *C*_lift_:*C*_drag, max_ are not adaptive but, instead, simply the secondary result of morphological adaptations to more dynamic, non-cruising flight.

The ratio of lift to drag is indicative of the overall glide performance of an aerofoil in terms of the ‘shallowness’ of the glide angle that a bird is able to utilize. Conventional aerofoils may have *C*_lift_:*C*_drag, max_ values of 200 or more at high *Re* (Shyy et al., 1999). At lower *Re* values similar to those used here, *C*_lift_:*C*_drag, max_ of between 10 and 12.6 have been reported for birds trained to fly in wind tunnels (Henningsson & Hedenström, 2011). These *C*_lift_:*C*_drag_ ratios were, however, obtained primarily for specialist gliding and soaring species (Harris hawk, *Parabuteo unicinctus* (Temminck, 1824), laggar falcon, *Falco jugger* (Gray, 1834) and swift) and are not directly comparable to the present data as they include the lift and drag influences of the body, rectrices and the smooth fairing of the wings to the body. However, more comparable testing on static wings from similarly specialised species still reveal equally high maximum *C*_lift_:*C*_drag_ ratios (∼17), validating the static wing methodology (Withers, 1981). Non gliding-specialist birds similar to those tested here, however, have more conservative values of *C*_lift_:*C*_drag_, (3.8–6) (Withers, 1981). Such low *C*_lift_:*C*_drag_ may be more broadly representative of avian wings, particularly when tested in isolation from the body.

Given that the lift to drag ratio is fundamental to the gliding performance of the wing, the differences in *C*_lift_:*C*_drag, max_ between flight style groups are intuitive. For example, the lower *C*_lift_:*C*_drag, max_ of HF compared to UF species is expected and likely results from selection for shallow glide angles between bouts of flapping. Such features are not required by HF species, whose pointed wings are suited to fast flapping frequencies and high-speed performance during migration. The reasons underlying the differences between HF and FF species are less clear. Planform measures such as aspect ratio and span are similar between the groups tested, as are *C*_lift, max_ and *C*_drag, min_. The observed differences in glide performance may therefore result from more complex morphological measures such as wing profile and camber along the wing. These measures will differ in response to *U* and *α* as a result of wing twisting. Interspecific differences in these responses may contribute to the differing *C*_lift_:*C*_drag,_ profiles seen between FF and HF species.

Withers (1981) found correlations between *AR* and *C*_drag_, *C*_lift_ and *C*_lift_:*C*_drag_, but did not take flight style groups into account. The relationships found here between aerodynamic and morphological properties are complex and different between the flight style groups. *C*_lift, max_ increased with *AR* in UF and HF species as expected. The reason for the decrease in *C*_lift, max_ with *AR* in FF species is unclear, but may result from morphological parameters not measured here and which are therefore not controlled for in our statistical tests. For example, Withers (1981) found the magnitude of camber and the position of maximum thickness to be strongly correlated with *C*_lift, max_. It is possible that camber was lower in the high *AR* FF birds relative to that of the other flight style groups at the same *AR*. In reality, however, camber is very difficult to measure for inclusion as a controlled variable as it differs along the wing from root to tip and is modified (as mentioned previously) by a combination of airspeed and *α*. This issue could be resolved using photogrammetric techniques during wind tunnel testing. Another likely confounding factor is the difference in *S* at any given *AR*. *AR* increased with *S* in the UF species but decreased in the other flight style groups (Table 1). The result of this relationship between *AR* and *S* is that *C*_lift, max_ increases very rapidly with *S* in UF species compared to the other groups. *C*_drag, min_ was unaffected by *AR* suggesting a minimal role of induced drag in determining overall drag at low angles of attack and correspondingly of *C*_lift_. The lower *C*_drag, min_ for UF compared to other flight style groups at a given *AR* is therefore unrelated to *AR* and likely results from other morphological parameters such as camber and nose radius, with an additional effect of the slotted wing tips (Withers, 1981). Given that lifting of the primary feather tips is required for the slotted wing to fully function and feather deformation will have a strong influence on camber, feather morphological and structural parameters such as rachis stiffness may also influence differences in the drag performance observed between different flight style groups. Overall, *C*_lift_:*C*_drag, max_ changed with *AR* in a similar pattern to that of *C*_lift, max_, indicating that FF species do not benefit from increased glide performance as their *AR* increases. In contrast, UF species benefit from a relatively large increase in glide performance with increasing *AR*. Together these data suggest that adaptations towards flapping flight in FF birds negate the increases in *C*_lift_ (and therefore *C*_lift_:*C*_drag_) performance that are expected to result from increased *AR*.

The absolute values of lift and drag are likely to differ between the wings of free-flying birds and those in our experimental setup, primarily as a result of the severed proximal ends which will increase drag and may alter span-wise flow. In order to compare aerodynamic parameters between flight style groups and make inferences about the relevance of our data to free-flying birds, it was assumed that these effects were consistent across the wings tested. Although in reality this is very difficult to establish, it seems unlikely that the drag effects of the exposed proximal surface differ between species or flight style groups. Furthermore, these effects are likely to be small in comparison to the more significant effects of wing morphology that are of interest here. For example, the low *C*_drag_ values of the corvids are unlikely to have occurred as a result of lower drag at the proximal surface compared to other birds. Changes in span-wise flow resulting from slotted tips in these species may have influenced the measured aerodynamic parameters but such changes do not invalidate our methodology as they may be representative of the case for birds in the wild.

It was recently suggested that wing morphology is only weakly correlated with flight style categories and is, instead primarily phylogenetically determined (Wang & Clarke, 2015). In this study, we aimed to quantify the effects of wing shape associated with different flight style groups upon the passive aerodynamic properties of the wing. Although it seems likely that the observed aerodynamic differences represent relevant functional differences between flight style groups, in reality it is often difficult to untangle the relative effects of phylogeny and ecology. In addition, although it is appealing to infer that any differences between flight style groups are adaptive, there remains the possibility that the passive aerodynamic properties of the wings within different flight style groups are not wholly adaptive or relevant to the flight performance of the birds but are merely the product of shared evolutionary history within the groups or the product of alternative selection pressures upon wing shape. However, it seems unlikely that the observed morphological variation in avian wing shape is linked solely to phylogenetic inertia. Closely related species with different ecologies show differences in wing shape that are adaptive and clearly linked to flight style (Drovetski, 1996). Indeed, even within species basic wing morphological measures, as well as feather mechanical properties (De la Hera et al., 2010) are plastic and allow acclimation to differing aerodynamic requirements (Vanhooydonck et al., 2009). Here, there are clear morphological differences between flight style groups. Within these groups, birds are both closely related (with the exception of the woodpigeon in the FF group) and share common ecologies, and the data indicate that the morphologies associated with different flight styles are linked to differences in aerodynamic performance. The full adaptive nature of these aerodynamic performance differences, however, remains to be determined.

##  Supplemental Information

10.7717/peerj.2495/supp-1 Data S1 Raw aerodynamic data

## Additional Information and Declarations

### Data Availability

The following information was supplied regarding data availability: The raw data has been supplied as Data S1.
